# Machine Learning Decision Support for Detecting Lipohypertrophy With Bedside Ultrasound: Proof-of-Concept Study

**DOI:** 10.2196/34830

**Published:** 2022-05-06

**Authors:** Ela Bandari, Tomas Beuzen, Lara Habashy, Javairia Raza, Xudong Yang, Jordanna Kapeluto, Graydon Meneilly, Kenneth Madden

**Affiliations:** 1 Data Science Program University of British Columbia Vancouver, BC Canada; 2 Gerontology and Diabetes Research Laboratory University of British Columbia Vancouver, BC Canada; 3 Division of Endocrinology Department of Medicine University of British Columbia Vancouver, BC Canada; 4 Division of Geriatric Medicine Department of Medicine University of British Columbia Vancouver, BC Canada; 5 Centre for Hip Health and Mobility Vancouver, BC Canada

**Keywords:** insulin, lipoma, machine learning, diagnostic ultrasound, lipohypertrophy, diabetes, ultrasound images

## Abstract

**Background:**

The most common dermatological complication of insulin therapy is lipohypertrophy.

**Objective:**

As a proof of concept, we built and tested an automated model using a convolutional neural network (CNN) to detect the presence of lipohypertrophy in ultrasound images.

**Methods:**

Ultrasound images were obtained in a blinded fashion using a portable GE LOGIQ *e* machine with an L8-18I-D probe (5-18 MHz; GE Healthcare). The data were split into train, validation, and test splits of 70%, 15%, and 15%, respectively. Given the small size of the data set, image augmentation techniques were used to expand the size of the training set and improve the model’s generalizability. To compare the performance of the different architectures, the team considered the accuracy and recall of the models when tested on our test set.

**Results:**

The DenseNet CNN architecture was found to have the highest accuracy (76%) and recall (76%) in detecting lipohypertrophy in ultrasound images compared to other CNN architectures. Additional work showed that the YOLOv5m object detection model could be used to help detect the approximate location of lipohypertrophy in ultrasound images identified as containing lipohypertrophy by the DenseNet CNN.

**Conclusions:**

We were able to demonstrate the ability of machine learning approaches to automate the process of detecting and locating lipohypertrophy.

## Introduction

The most common dermatological complication of insulin therapy for glycemic control in diabetes is lipohypertrophy, which has a prevalence ranging from approximately 25% to 65% in the literature [1,2]. These lesions are characterized by fibrosis, decreased vascularity, and adipose hypertrophy [3] and are likely due to both inflammation and the trophic properties of insulin [4]. These lesions have clinical effects that reach far beyond the skin—some previous works have shown that lipohypertrophy alters insulin absorption resulting in poor glycemic control and high glycemic variability in persons with diabetes [5-7]. Avoidance of lipohypertrophic sites has also shown to improve glycated hemoglobin levels, and current practice recommends the evaluation of these lesions based on either visual inspection or palpation [8,9]. More recent findings have developed clear criteria for detecting lipohypertrophy with ultrasound and have shown that approximately half of these lesions are not detectable by palpation [10,11]. These findings have led to the suggestion that bedside ultrasound can be used as an adjunct to palpation [10], but there are significant barriers to implementing this in standard diabetes clinics since ultrasound imaging is only familiar to and implemented by a small group of diabetes educators or physicians.

The development of machine learning techniques to predict masses in ultrasound images has been an ongoing effort in clinical practice for the past few decades. To assist physicians in diagnosing disease, many scholars have implemented techniques such as regression, decision trees, Naive Bayesian classifiers, and neural networks on patients’ ultrasound imaging data [12]. Further, many studies involving ultrasound images have attempted to preprocess the images to extract features. Previous work by Chiao et al [13] has demonstrated that the use of convolutional neural networks (CNNs) with ultrasound images is better than radiomic models in predicting breast cancer tumors [13]. Other recent work has shown success in classifying liver masses into 1 of 5 categories with 84% accuracy, using a CNN model [14]. Recent work looking into the use of various complex image augmentation approaches has shown that the use of generative adversarial networks to generate images to enlarge the data set improve the performance of the eventual model [15], and many such studies [16,17] have confirmed that minimal transformations such as flipping images can result in a higher prediction accuracy.

In an effort to improve the accessibility and efficiency of this method of detection, we have, as a proof of concept, developed a supervised machine learning algorithm to detect lipohypertrophy in ultrasound images using a CNN and a web-based application to deploy the trained models and make accurate predictions on the presence or absence of lipohypertrophy in ultrasound images.

## Methods

### Recruitment

All images were obtained from research participants who were enrolled in a diabetes education program at an academic center and who had an unknown lipohypertrophy status between July 2015 and March 2017 as part of a previous study of this condition [10]. All research participants were above 19 years of age, had a diagnosis of type 1 or type 2 diabetes mellitus, and were currently being treated with a minimum of 1 insulin injection daily or an insulin pump for at least 2 years. Participants were excluded if they were prescribed a systemic glucocorticoid, glucagon-like peptide-1 agonist, or if they had a nonlipodystrophic dermatological condition extending to the insulin injection site area. Each image was categorized as positive (lipohypertrophy present) or negative (no lipohypertrophy present) by a radiologist as per previously published criteria in a blinded fashion [10]. Ultrasound images were obtained in a blinded fashion using a portable GE LOGIQ *e* machine with an L8-18I-D probe (5-18 MHz; GE Healthcare).

### Ethical Considerations

All research participants gave written consent, and our study protocol received approval by the Human Subjects Committee of the University of British Columbia (H20-03979).

### Data Splits

Before beginning any model training, the data were split into train, validation, and test splits of 70%, 15%, and 15%, respectively, followed by some preprocessing steps of manually removing borders from the nonannotated versions of the images. We included all different types of diabetes as 1 set and did not differentiate between patients when splitting, as the histology of these lesions has been found to be independent of the source of insulin or mode of administration [18,19]. In fact, insulin-induced lipohypertrophy does not show any histological specificity, closely resembles hypertrophic cellulite [20], and appears identical to fat nodules due to other etiologies such as corticosteroids [21] or electromagnetic fields [22]. The lesions have been shown to be due to the direct result of the hypertrophic effects of administered insulin with no evidence for a pathogenic role for the insulin antibodies found in type 1 diabetes [23].

### Image Transformation and Model Development

**Figure 1:**
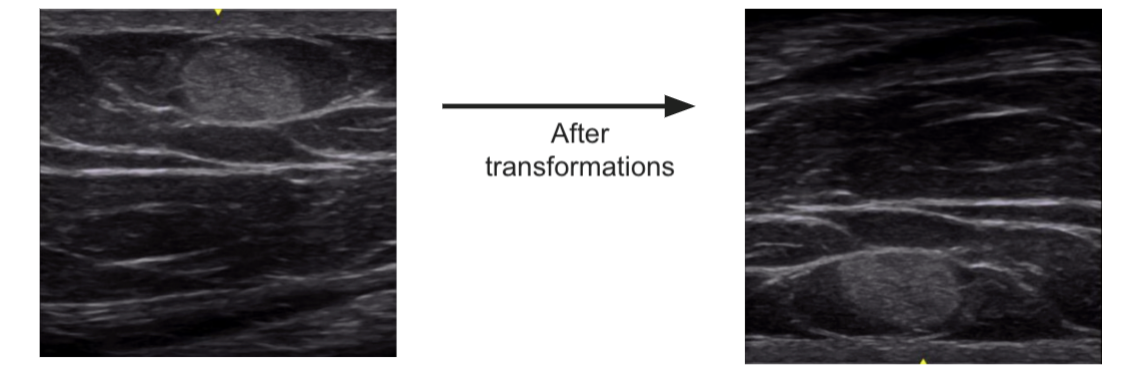
Final image transformations included random vertical and horizontal flipping and random brightness and contrast adjustment.

Given the small size of the data set, image augmentation techniques were used to expand the size of the training set and improve the model’s generalizability. A variety of classic transformations [16,17] were tested, and the model’s performance on these augmented data sets were documented at this stage (Figure 1). The augmenting transformations that led to the best performance were adding random vertical and horizontal flipping, randomly changing the brightness between –0.1 to 0.1, and randomly changing the contrast between 0 and 1, each with a probability of 50%. The images in the data set varied in size from 300300 up to 460500. As a result, after the above transformations, all images were resized to a standard common denominator of 300300 pixels by cropping. An example of a transformed image is shown in Figure 1. The augmented data is then used to train a CNN model using transfer learning, a technique using pretrained models on thousands of images, which then allows for retraining of the entire network with our comparatively smaller data set. Based on our literature review, the transfer learning architectures we chose to investigate were the following: VGG16, ResNet50, DenseNet169, and InceptionV3 [24]. Each model was incorporated into our small data set, trained in separate experiments using techniques to optimize the parameters of the model to maximize its ability to learn. To compare the performance of different architectures, the team considered the accuracy and recall scores of the models when tested on our test set.

### Object Detection

In addition, we wanted to implement object detection into our pipeline, giving users the opportunity to visually identify the location of lipohypertrophy being detected by our model. To implement object detection using a popular framework called YOLOv5 [25,26], the team created bounding boxes around the location of the lipohypertrophy masses on the positive training images using the annotated ultrasound images as a guide. Next, using the YOLOv5 framework, the YOLOv5m model was trained for 200 epochs with an image size of 320320 pixels (as this was what the Application Programming Interface allowed) and a batch size of 8.

## Results

**Figure 2:**
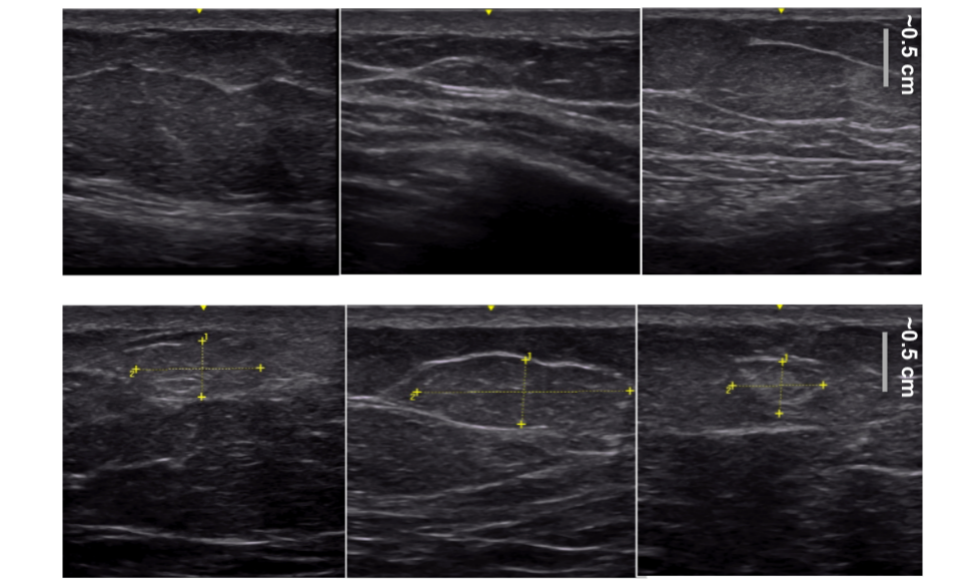
Some examples of images found in our data set. The top row displays negative images (no lipohypertrophy present) and the bottom row displays positive images (lipohypertrophy present) where the yellow annotations indicate the exact area of the mass. The yellow annotations are only for the reader; the images that the model was trained on were unmarked with no yellow annotations.

Our images were obtained from a total of 103 participants, of whom 8% were diagnosed with type 1 and 92% were diagnosed with type 2 diabetes (Table 1). Our data set included 218 negative images (no lipohypertrophy present) and 135 positive images (lipohypertrophy present). Examples are shown in Figure 2.

Each of the potential models (VGG16, ResNet50, DenseNet169, and InceptionV3) were investigated by training them in separate experiments, using our augmented data set.

**Table 1:** Research participant characteristics (N=103).

Characteristics	Values
Age (years), mean (SE)	75.0 (11.8)
BMI (kg/m^2^), mean (SE)	28.3 (6.1)
Participant with type 1 diabetes, n	8
Number of years on insulin, mean (SE)	9.4 (11.5)
Duration of diabetes (years), mean (SE)	20.7 (6.1)
Glycated hemoglobin (%), mean (SE)	8.0 (1.1)
Total daily dose (units), mean (SE)	48.6 (42.9)
Daily doses, n (range)	2 (1-6)

As shown in Table 2, all models were able to achieve accuracy scores higher than 0.60 when tested on a holdout sample. When comparing performance of the various models, DenseNet demonstrated the highest accuracy score (0.76), the highest recall or sensitivity score (0.76), and the highest specificity score (0.49), indicating an overall better performance than Inception, VGG16, or ResNet. In addition to better performance, DenseNet also demonstrated a relatively small computational size (30 MB) compared to the other models (Inception, 100 MB; ResNet, 99 MB; VGG16, 547 MB).

**Figure 3:**
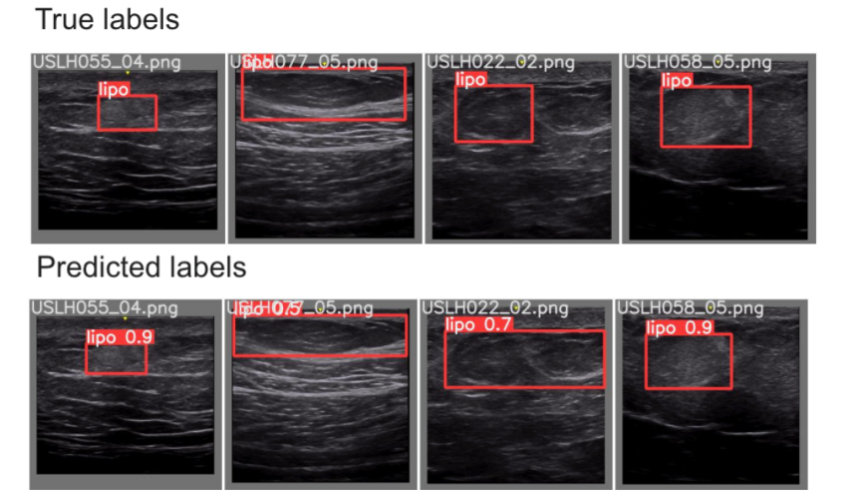
Our final object detection model results on a test sample reveals promising outcomes. The top row indicates the true location of lipohypertrophy, and the bottom row indicates where the model thinks the lipohypertrophy is. The number on the red box indicates the model’s confidence.

**Figure 4:**
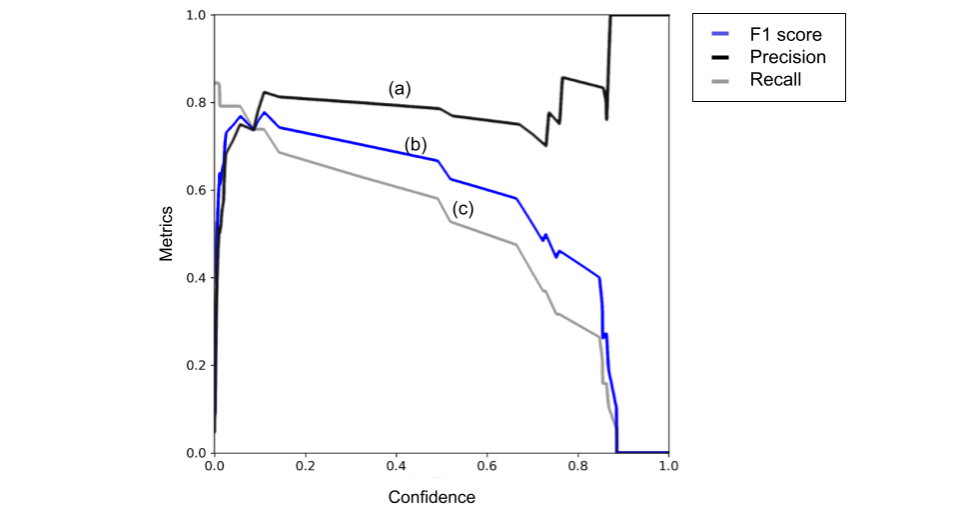
Our results from the YOLOv5m object detection model showcase a successful initial attempt, as shown by our precision (a). Our best F1 score (b) is around 0.78 with a confidence value of about 0.4109. Any higher confidence value causes our recall (c) to suffer dramatically, which was the focus of our optimization.

With respect to object detection implementation, the YOLOv5m model was able to identify the specific location of lipohypertrophy in test cases, as demonstrated in Figure 3. In order to help a clinician verify the results of our models, YOLOv5m was able to accurately create bounding boxes around lipohypertrophy sites in ultrasound images. As shown in Figure 4, YOLOv5m demonstrated an F1 score of 0.78 at a confidence value of 0.41.

All 4 models (ResNet, VGG16, Inception, and DenseNet) were tested on a holdout sample to produce these accuracy, recall or sensitivity, and specificity results.

**Table 2:** Model accuracy scores, recall or sensitivity scores, and specificity scores.

Model	Accuracy scores	Recall or sensitivity scores	Specificity scores
DenseNet	0.76	0.76	0.49
Inception	0.74	0.52	0.33
VGG16	0.65	0.19	0.12
ResNet	0.61	0	0

## Discussion

### Principal Results

As a proof of concept, we were able to demonstrate the ability of a supervised machine learning algorithm to detect lipohypertrophy on ultrasound images using a CNN, and we were able to deploy this algorithm though a web-based application to make accurate predictions on the presence or absence of lipohypertrophy in ultrasound images obtained at the point of care. The DenseNet transfer learning architecture outperformed the other architectures tested, suggesting this would be the most appropriate choice to automate the process of detecting and locating lipohypertrophy, a common dermatological complication of insulin injections.

### Comparison With Prior Works

Prediction of masses in ultrasound images using machine learning techniques has been an ongoing effort in clinical practice for the past few decades. To assist physicians in diagnosing disease, many scholars have implemented techniques such as regression, decision trees, Naive Bayesian classifiers, and neural networks on patients’ ultrasound imaging data [12]. Further, similar to this study, many investigators have used preprocessing techniques to extract features. In fact, Chiao et al [13] demonstrated that CNNs using ultrasound images perform better than other methods (such as radiomic models) in predicting breast cancer tumors. Another recent study showed considerable success in classifying liver masses into 1 of 5 categories with 84% accuracy, using a CNN mode [14]. To our knowledge, this is the first attempt to use CNN techniques to automate the detection of lipohypertrophy, demonstrating the considerable performance of our DenseNet model both in terms of test accuracy and recall (Table 2).

Recent research has delved into various complex image augmentation techniques to generate images [15]; we also found that traditional transformations managed to improve model performance, congruent with the results of this study. Furthermore, other studies [16,17] also confirmed that minimal transformations such as flipping the images led to higher prediction accuracy in their application. DenseNet has also proved successful in similar deep learning applications using small data sets [27], which we suspect is due to its ability to reduce the parameters in a model.

### Limitations

Although our project has demonstrated in principle that machine learning can be used to detect lipohypertrophy, there are some key limitations that should be addressed before it can be used in a clinical setting. Given the small size of our data set, more images need to be incorporated into the model before it can be used to direct patient care. Besides, even after the addition of new images, an auditing process should also be developed to ensure that our machine learning model does not propagate any biases that could cause harm to specific patient populations.

### Conclusions

Previous clinical studies of lipohypertrophy have demonstrated quite a high prevalence of this condition (greater than half). More importantly, they have demonstrated a significant burden of subclinical lesions in patients with diabetes [10]. This is clinically important both due to the alterations in insulin absorption with injection proximate to a lipohypertrophic lesion [5-7] and the fact that the only treatment for this condition is avoidance [28]. Although our proof-of-concept study was limited by the fact that our model was based on a small number of images, we have successfully demonstrated the development of a model that can automatically detect lipohypertrophy in patients with diabetes. Although more work needs to be done, future studies of models developed on larger image data sets could allow for the development of a rapid, noninvasive, bedside test for subclinical lipohypertrophy that could easily be used by health care professionals unfamiliar with the use of ultrasound technology.

## Abbreviations

CNN: convolutional neural network
